# Rose hip and its constituent galactolipids confer cartilage protection by modulating cytokine, and chemokine expression

**DOI:** 10.1186/1472-6882-11-105

**Published:** 2011-11-03

**Authors:** Joseph Schwager, Ulrich Hoeller, Swen Wolfram, Nathalie Richard

**Affiliations:** 1 DSM Nutritional Products Ltd., Department of Human Nutrition & Health, P.O. Box 2676, CH-4002 Basel, Switzerland

## Abstract

**Background:**

Clinical studies have shown that rose hip powder (RHP) alleviates osteoarthritis (OA). This might be due to anti-inflammatory and cartilage-protective properties of the complete RHP or specific constituents of RHP. Cellular systems (macrophages, peripheral blood leukocytes and chondrocytes), which respond to inflammatory and OA-inducing stimuli, are used as *in vitro *surrogates to evaluate the possible pain-relief and disease-modifying effects of RHP.

**Methods:**

(1) Inflammatory processes were induced in RAW264.7 cells or human peripheral blood leukocytes (PBL) with LPS. Inflammatory mediators (nitric oxide (NO), prostaglandin E_2 _(PGE_2_) and cytokines/chemokines) were determined by the Griess reaction, EIA and multiplex ELISA, respectively. Gene expression was quantified by RT-PCR. RHP or its constituent galactolipid, GLGPG (galactolipid (2*S*)-1, 2-di-*O*-[(9*Z*, 12*Z*, 15*Z*)-octadeca-9, 12, 15-trienoyl]-3-*O*-β-D-galactopyranosyl glycerol), were added at various concentrations and the effects on biochemical and molecular parameters were evaluated. (2) SW1353 chondrosarcoma cells and primary human knee articular chondrocytes (NHAC-kn) were treated with interleukin (IL)-1β to induce *in vitro *processes similar to those occurring during *in vivo *degradation of cartilage. Biomarkers related to OA (NO, PGE_2_, cytokines, chemokines, metalloproteinases) were measured by multiplex ELISA and gene expression analysis in chondrocytes. We investigated the modulation of these events by RHP and GLGPG.

**Results:**

In macrophages and PBL, RHP and GLGPG inhibited NO and PGE_2 _production and reduced the secretion of cytokines (TNF-α, IFN-γ, IL-1β, IL-6, IL-12) and chemokines (CCL5/RANTES, CXCL10/IP-10). In SW1353 cells and primary chondrocytes, RHP and GLGPG diminished catabolic gene expression and inflammatory protein secretion as shown by lower mRNA levels of matrix metalloproteinases (MMP-1, MMP-3, MMP-13), aggrecanase (ADAMTS-4), macrophage inflammatory protein (MIP-2, MIP-3α), CCL5/RANTES, CXCL10/IP-10, IL-8, IL-1α and IL-6. The effects of GLGPG were weaker than those of RHP, which presumably contains other chondro-protective substances besides GLGPG.

**Conclusions:**

RHP and GLGPG attenuate inflammatory responses in different cellular systems (macrophages, PBLs and chondrocytes). The effects on cytokine production and MMP expression indicate that RHP and its constituent GLGPG down-regulate catabolic processes associated with osteoarthritis (OA) or rheumatoid arthritis (RA). These data provide a molecular and biochemical basis for cartilage protection provided by RHP.

## Background

A primary feature of arthritis, in particular osteoarthritis (OA), is the degradation and erosion of the extracellular matrix (ECM) in cartilage. The preceding alterations of collagen and proteoglycan implicate the activation of enzymatic systems, *i.e*. matrix metalloproteinase (MMPs) and aggrecanase (*e.g*. a disintegrin and metalloproteinase with thrombospondin type I motif, ADAMTS) [1,2]. Specifically, MMP-1, MMP-3 and MMP-13 cleave ECM collagen [3-5]. Interleukin (IL)-1β is considered a key catabolic factor that induces ECM degradation (reviewed in [6]). IL-1β has multiple effects on the expression of chondrocyte genes and affects matrix enzymes, chemokines and cytokines. Some of these effects are opposed by transforming growth factor beta (TGF-β) or bone morphogenic protein (BMP)-2 [7]. Nitric oxide (NO) has been identified as another agent in OA (reviewed in [8,9]): the expression of inducible nitric oxide synthase (iNOS) and the production of NO correlate with patho-physiological changes in chondrocytes [10-13]. The importance of chemokines in OA was highlighted by the observation that numerous chemokines and their receptors were massively induced by IL-1β in chondrocytes [7,14-16]. This underscores the importance of cell recruitment during inflammatory processes in OA.

Natural substances may attenuate the onset and progression of OA. Clinical studies have demonstrated a beneficial effect of rose hip powder (RHP) in the treatment of OA [17-19] (for reviews see [20,21]). The underlying bioactive constituents of RHP remain elusive, although its known constituents such as ascorbic acid, polyphenols, flavonoids and unsaturated fatty acids presumably contribute to alleviate OA. More specifically, GLGPG, a galactolipid isolated from RHP, inhibited chemotaxis of neutrophils [17,18]. Also, RHP extracts and unsaturated fatty acids thereof inhibited cyclooxygenase (COX)-1 and COX-2 activity [22,23] and could partially account for the efficacy of RHP in the treatment of OA. Yet, RHP and/or its constituents have not been directly tested in chondrocytes. In this study, the effects of RHP and one of its constituent galactolipid, GLGPG, have been evaluated on (1) the production of inflammatory mediators by macrophages and peripheral blood leukocytes, and (2) anabolic and catabolic processes in chondrocytes.

## Methods

### Reagents

RHP, prepared from dried *Rosa canina *fruits of a selected cultivar, was obtained from Hyben Vital, Langeland, Denmark. GLGPG (galactolipid (2*S*)-1, 2-di-*O*-[(9*Z*, 12*Z*, 15*Z*)-octadeca-9, 12, 15-trienoyl]-3-*O*-β-D-galactopyranosyl glycerol, abbreviated as GOPO in [18]) was isolated from a lipophilic extract prepared from leaves of *Valeriana locusta *or from RHP (performed by AnalytiCon Discovery, Potsdam, Germany). Compounds were dissolved in DMSO and added to the culture medium concomitantly with the stimulating agent. Final DMSO concentration in culture medium in all treatments .was 0.5%. *E. coli* LPS (serotype 055:B5) and fetal bovine serum (FBS) were from Sigma (Saint-Louis, MO). RPMI 1640, DMEM, 2-mercaptoethanol and MEM non-essential amino acids (NEAA) were from Invitrogen (Carlsbad, CA). Human IL-1β and recombinant interferon-γ (IFN-γ) were from PeproTech EC (London, UK).

### Cell culture

Murine RAW264.7 macrophage cells were from ATCC (Manassas, VA) and cultured in DMEM supplemented with 50 U/mL penicillin, 50 μg/mL streptomycin, 0.1 mM NEAA (DMEM-C) and 10% FBS. Cells were seeded into 12-well or 96-well plates at 1 and 0.05 × 10^6 ^cells per well, respectively, and used after 2 days of pre-culture. Cells were starved for 18 h in DMEM-C containing 0.25% FBS before the start of treatment and stimulated with LPS (1 μg/mL) for 4-24 h in phenol red-free DMEM-C containing 0.25% FBS.

Peripheral blood leukocytes (PBL) were obtained from healthy male and female donors (Blood Donor Service, University Hospital, Basel, Switzerland). Human primary cell protocols were approved by the Swiss Federal Office of Public Health (No. A050573/2 to J. Schwager). Erythrocytes were removed by the Dextran sedimentation procedure [24]. Cell viability was determined by the Trypan Blue exclusion test and exceeded 95%. PBL (at 3-8 × 10^6 ^cells/mL) were cultured in phenol-red free RPMI 1640, supplemented with 0.25% FBS, 0.1 mM NEAA, 50 U/mL penicillin, 50 μg/mL streptomycin and 5 × 10^-5 ^M 2-mercaptoethanol. Cells were stimulated with LPS (100 ng/mL) and IFN-γ (20 U/mL) for 2-24 h.

SW1353 chondrosarcoma cells were from ATCC and cultured in DMEM-C containing 10% FBS. Cells were seeded into 6-well plates at 0.5 × 10^6 ^cells per well. Sub-confluent cell monolayers were washed and incubated overnight in DMEM-C containing 0.25% FBS and 0.2% lactalbumin hydrolysate (Bacto™ LC, Becton Dickinson, Franklin Lakes, NJ). Cells were activated with 10 ng/mL IL-1β in phenol-red free DMEM-C supplemented with 0.25% FBS and 0.2% lactalbumin hydrolysate in the presence of increasing concentrations of test compounds for 4-24 h. Batches of normal human articular chondrocytes from knee (NHAC-kn) obtained from different individuals were from Lonza and cultured in chondrocyte growth medium (Lonza, Wakersville, MD). For experiments NHAC-kn were used at passage 3 to 6. Cells were seeded into 6-well plates at 0.5 × 10^6 ^cells per well and activated with IL-1β (10 ng/mL) for 4-24 h.

Cells were lysed in RLT buffer (Qiagen) after 2-4 h of culture and total RNA was extracted. Culture supernatants were harvested after 24 h of culture and stored at -80°C until use for analysis.

### RNA isolation, cDNA synthesis and RT-PCR

Total RNA was isolated using the RNeasy Mini Kits (Qiagen, Hilden, Germany) as previously described [25]. RNA quality and quantity was assessed by Nanodrop^® ^ND-1000 and evaluated by the ND-1000 3.2.1 software (Witec AG, Littau, Switzerland).

Total RNA was transcribed into first strand cDNA using the Superscript™ First-Strand Synthesis System for RT-PCR from Invitrogen (Carlsbad, CA) [25]. Real-time PCR analysis was performed using the ABI PRISM^® ^7700 Sequence Detection System or the ABI 7900HT Fast Real-Time PCR System (Applied Biosystems [ABI], Foster City, CA). Primers and probes were designed with the Primer Express™ software purchased from ABI. PCR was performed using the Taqman^® ^universal PCR Master Mix (ABI). 18S rRNA primers and probes were used as internal standards. Relative gene expression quantification was performed by subtracting threshold cycles (C_T_) for ribosomal RNA from the C_T _of the targeted gene (ΔC_T_). Relative mRNA levels were then calculated as 2^-ΔΔCT^, where ΔΔC_T _refers to the ΔC_T _of unstimulated minus treated cells. The values were obtained from at least three independent series of experiments, in which each treatment was performed in duplicate with each being analyzed twice in RT-PCR.

### Multiparametric analysis of cytokines, chemokines and interleukins

Multiparametric kits were obtained from BIO-RAD Laboratories (Hercules, CA) and used in the LiquiChip Workstation IS 200 (Qiagen, Hilden, Germany). The data were evaluated with the LiquiChip Analyser software (Qiagen). In all experimental series, the proteins secreted into the culture supernatants were determined.

### Measurement of nitric oxide and PGE_2 _determination

The concentration of NO in culture supernatants was measured using the Griess Reaction [26]. Secreted PGE_2 _was determined by Enzyme Immuno Assay (EIA) (Cayman Chemicals, Ann Harbor, WI).

### Statistical analysis

Data were obtained from at least three independent series of experiments and presented as means +/- SD (in ELISA). *p *values < 0.05 (calculated by Student's t test or one way ANOVA) were considered statistically significant.

## Results

### Rose hip and GLGPG inhibit the production of inflammatory mediators by murine macrophages

Macrophages represent a cellular model to identify effects of substances on inflammatory processes including those associated with arthritis. RAW264.7 cells responded to LPS-stimulation by producing inflammatory mediators (*e.g*. NO and PGE_2_). In unstimulated cells, test substances alone did not modulate the secretion of inflammatory mediators. In LPS-treated cells, RHP reduced NO production and PGE_2 _secretion at IC_50 _values of 797 mg/L and 594 mg/L, respectively (Table 1). Similarly, GLGPG significantly inhibited the NO production (IC_50 _values of 28.6 mg/L), whereas PGE_2 _secretion was diminished by 14 ± 9% at 38.7 mg/L (*i.e*. highest concentration tested). The data are consistent with the described effects of rose hip constituents on the activity of COX isozymes [22,23]. At the investigated concentrations, RHP or GLGPG did not impair cell viability as determined by LDH release (data not shown).

**Table 1:** Effect of RHP and GLGPG on NO and PGE_2 _production in RAW264.7 cells

Substance	IC_50 _± SEM (NO) [mg/L]	IC_50 _± SEM (PGE_2_) [mg/L]
GLGPG^a^	28.6 ± 4.6 (N = 6)	> 38.7^c ^(N = 6)

RHP^b^	796.9 ± 36.9 (N = 15)	594 ± 43 (N = 14)

LPS-stimulated cells were cultured for 24 h with 31.3 - 1000 mg/L RHP, or 1.2 - 38.7 mg/L GLGPG. The concentration of NO and PGE_2 _was determined in culture supernatants and the IC_50 _values (± SEM, standard error of the mean) were computed. N, number of independent experimental series. At the conditions described in Materials and Methods, unstimulated and LPS-stimulated cells produced < 0.125 μM nitrite and 25.4 ± 1.2 μM, respectively.

^a^GLGPG: galactolipid (2*S*)-1, 2-di-*O*-[(9*Z*, 12*Z*, 15*Z*)-octadeca-9, 12, 15-trienoyl]-3-*O*-β-D-galactopyranosyl glycerol; MW 774

^b^From Hyben Vital

^c ^Highest concentration of GLGPG tested. At 38.7 mg/L GLGPG, PGE_2 _production was inhibited by 14 ± 9%.

**Figure 1:**
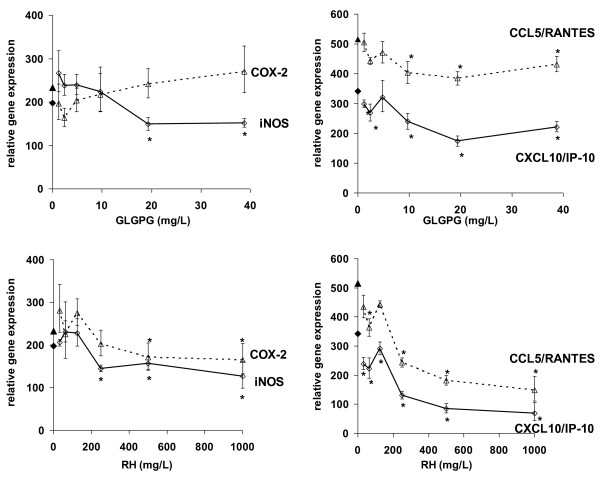
**Effect of RHP and GLGPG on gene expression in RAW264.7 cells**. Expression levels of COX-2, iNOS, CCL5/RANTES and CXCL10/IP-10 in RAW264.7 cells stimulated with LPS for 4 h in the presence of 1.2 - 38.7 mg/L (*i.e *1.25 -50 μmol/L) of GLGPG (upper panels) or 31.3 - 1000 mg/L RHP (lower panels). Relative gene expression refers to 2^-ΔΔCT ^(see Materials and Methods). Filled symbols on the y-axis indicate the values observed in LPS-stimulated cells (without test substances). * *p *< 0.05.

LPS-stimulation of RAW264.7 elicited the expression of inflammatory genes. Most (*e.g*. genes for TNF-α, COX-2, iNOS, IL-1α, CCL5/RANTES, CXCL10/IP-10) were drastically up-regulated (Table 2 and Additional File 1). We had determined in previous experiments that at 2, 4 and 8 h of stimulation, LPS-responding genes were significantly up-regulated (data not shown); at 4 h both early- and late-responding genes were significantly expressed. At this point in time, the iNOS mRNA levels were concentration-dependently reduced by GLGPG and RHP (Figure 1). COX-2 mRNA levels were not affected, but those of prostaglandin E synthase (PGES), which converts PGH_2 _to PGE_2_, were lowered (Table 2 and Additional File 1). Among murine cytokines and chemokines, CCL5/RANTES and CXCL10/IP-10 were robustly down-regulated by RHP and GLGPG. The expression of MMP-9 was also significantly reduced by RHP and less profoundly by GLGPG. Remarkably, expression of the anti-inflammatory IL-10 was increased by both substances. Macrophage genes encoding transcription factor (TF) of the NF-κB signaling pathway (*i.e*. NF-κB1, NF-κB49, NF-κBp65, I-κBα) were up-regulated by LPS (Table 2 and Additional file 1). RHP significantly reduced all TFs towards pre-stimulation values or below. GLGPG had less robust effects on those factors and markedly reduced only NF-κB49. Thus, the test compounds modulated gene expression at the transcriptional level *via *elements of the NF-κB pathway.

**Table 2:** Modulation of gene expression in RAW264.7 cells by RHP and GLGPG

Gene	C_T_^a^	LPS	LPS + RHP (250 mg/L)		LPS + GLGPG (9.7 mg/L)	
		fold change	fold change	*p *value^b^	fold change	*p *value^b^
TNF-α	22.9	**29.6**	**21.8**	0.0001	**20.4**	0.002
COX-2	27.5	**247**	**203.3**	0.347	**217**	0.660
PGES	33.0	**2.2**	**1.6**	0.0107	**2.0**	0.214
iNOS	29.9	**197**	**239.4**	0.041	**224**	0.357
						
IL-1α	33.7	**22081**	**8128**	0.0115	**18501**	0.087
IL-10	38.3	**66.8**	**176**	< 0.0001	**78.6**	0.074
						
CCL5/RANTES	29.7	**515**	**121**	< 0.0001	**403**	0.003
CXCL10/IP-10	27.2	**342**	**65.4**	0.0001	**239**	0.021
						
NF-κB1	24.8	**6.3**	**2.1**	0.0002	**6.0**	0.366
NF-κB49	25.3	**4.1**	**1.5**	0.0028	**3.7**	0.028
NF-κBp65	23.6	**1.2**	**0.4**	0.0045	**1.1**	0.126
I-κBα	24.4	**5.5**	**2.0**	0.0004	**5.7**	0.391
						
CD14	24.9	**1.0**	**0.5**	0.198	**1.4**	0.012
MMP-9	29.0	**6.8**	**3.0**	0.0493	**7.8**	0.145

LPS-stimulated cells were cultured with 31.3 - 1000 mg/L RHP or 1.2 - 38.7 mg/L of GLGPG for 4 h and gene expression was quantified by RT-PCR. Fold changes were calculated as indicated in Materials and Methods. Only the effects of 250 mg/L RHP and 9.7 mg/L GLGPG are shown.

^a^C_T _= cycle threshold (C_T _< 22 = high basal gene expression; 23 < C_T _< 30 = intermediate basal gene expression; C_T _> 30 = low basal gene expression).

^b^*p *value = significance value between (LPS)- and (LPS + compound)-treatment.

### Effect of RHP and GLGPG on peripheral blood leukocytes

**Figure 2:**
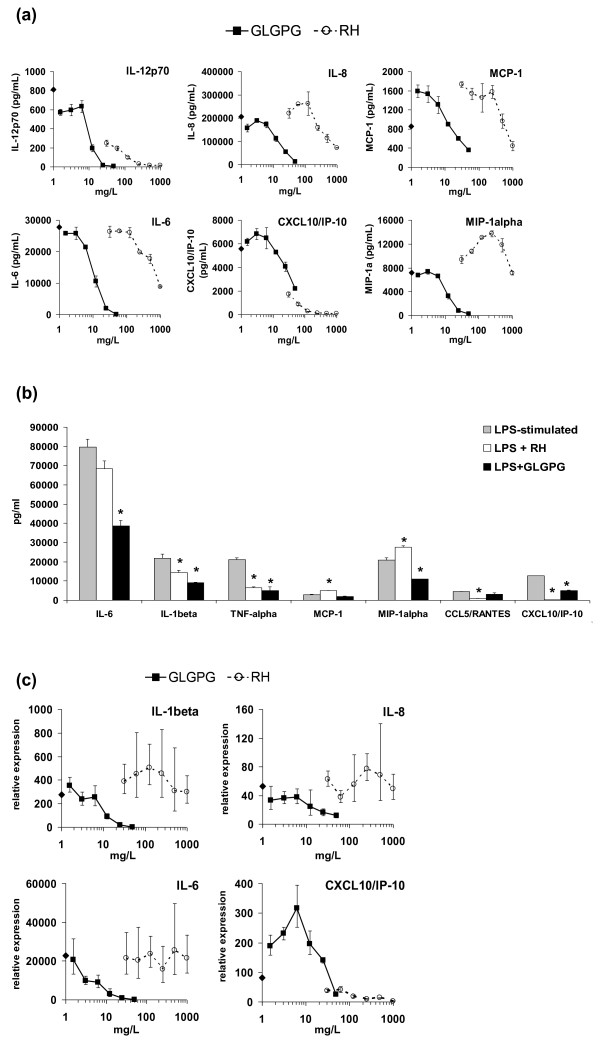
**Effect of RHP and GLGPG on inflammatory mediators in PBL**. **(a) **Proteins secreted by PBLs during LPS/IFN-γ stimulation for 24 h without (symbol on the y axis) or with 31.3 - 1000 mg/L RHP or 1.2 - 38.7 mg/L of GLGPG. **(b) **Effects of RHP (250 mg/L) and GLGPG (9.7 mg/L) on the secretion of interleukins and chemokines by LPS/IFN-γ stimulated PBL that were cultured for 24 h. Asterisks indicate statistical significant differences in comparison to LPS-stimulated cells (* *p *< 0.05). **(c) **Expression levels of interleukins (IL-1β and IL-6) and chemokines (IL-8, CXC10/IP-10) in LPS/IFN-γ stimulated PBL cultured for 2 h with 31.3 - 1000 mg/L RHP or 1.2 - 38.7 mg/L of GLGPG. The symbol on the y-axis indicates the LPS/IFN-γ -induced increase in mRNA levels, in the absence of test compounds.

Orally absorbed bioactive compounds reach target tissues *via *the vascular system, where they might exert effects on peripheral blood leukocytes (PBL). Therefore, the effects of RHP and GLGPG on LPS/IFN-γ -activated PBL was investigated. The secretion of chemokines or interleukins was modulated by RHP and GLGPG in an idiosyncratic pattern (Table 3; Figure 2a-b and Additional file 2). RHP significantly reduced the production of CXCL10/IP-10 and CCL5/RANTES; the levels of other chemokines were unaltered or only affected at the highest tested RHP concentrations (Figure 2a). GLGPG reduced MCP-1 and MIP-1α secretion. It did, however, not match the effect of RHP on CXCL10/IP-10 and CCL5/RANTES production. RHP had robust effects on secretion levels of (pro-inflammatory) IL-12p70 and (anti-inflammatory) IL-10, while it modulated IL-1β and IL-6 less profoundly. This contrasted with the effects of GLGPG, which significantly diminished IL-1β and IL-6 (Figure 2b). TNF-α and IFN-γ were concentration-dependently diminished by both RHP and GLGPG (Figure 2b, Table 3). Furthermore, in PBL RHP increased the expression of GM-CSF, an anabolic factor for chondrocytes [27]. Next, expression levels of inflammatory mediators were determined in human PBL. In most cases, RHP and GLGPG exerted effects on mRNA levels that paralleled those described for the secreted proteins (Table 4, Figure 2c and Additional file 3). In particular, RHP robustly down-regulated mRNA levels of IL-1α, TNF- α and CXCL10/IP-10, but not those of *e.g*. IL-6, CCL5/RANTES, COX-2, MIP-2 or MIP-3α. Other genes that are involved in the production of eicosanoids (*e.g*. 5-lipoxygenase) or participate in ECM remodeling (*e.g*. MMP-9) were not modulated by the test compounds. The results infer that the main effects of the natural substances were at the transcriptional rather than the post-transcriptional level. A notable exception was CCL5/RANTES, where mRNA levels were barely up-regulated by LPS/IFN-γ stimulation, while the secretion of CCL5/RANTES was impaired by the natural substances.

**Table 3:** Effect of RHP and GLGPG on the production of chemokines and cytokines in human PBL

Protein	Ratio^a^	LPS/IFN-γ	LPS/IFN-γ + RHP (250 mg/L)		LPS/IFN-γ + GLGPG (9.7 mg/L)	
		pg/mL ± SD	pg/mL ± SD	*p *value^b^	pg/mL ± SD	*p *value^b^
Eotaxin	28	84 ± 2	62 ± 6	*0.037*	62 ± 3	*0.013*
MCP-1	497	2940 ± 226	4990 ± 71	*0.007*	1880 ± 368	*0.074*
MIP-1β	169	90900 ± 1838	109009 ± 1823	*0.005*	47550 ± 3465	*0.004*
MIP-1α	2092	21079 ± 1131	27510 ± 849	*0.023*	10950 ± 71	*0.006*
IL-8	794	325103 ± 12738	433040 ± 122728	*0.059*	267052 ± 36770	*0.255*
CCL5/RANTES	14	4470 ± 57	1011 ± 55	*< 0.001*	3210 ± 750	*0.067*
CXCL10/IP-10	47	12650 ± 16	150 ± 16	*0.002*	5025 ± 148	*0.005*
IL-1β	> 2000	21900 ± 2121	14350 ± 1344	*0.051*	9195 ± 106	*0.014*
IL-6	> 2000	79650 ± 4031	68450 ± 4031	*0.083*	38604 ± 2828	*0.005*
IL-12(p70)	> 200	295 ± 2	19 ± 2	*0.002*	69 ± 17	*0.006*
IL-10	67	233 ± 8	382 ± 3	*0.002*	143 ± 47	*0.118*
TNF-α	> 2000	21107 ± 990	6595 ± 290	*0.003*	5105 ± 1775	*0.008*
IFN-γ	672	2065 ± 106	226 ± 18	*0.002*	475 ± 2	*0.002*
GM-CSF	158	148 ± 5	190 ± 47	*0.329*	54 ± 2	*0.002*

LPS/IFN-γ -stimulated cells were cultured with 31.3 - 1000 mg/L RHP or 1.2 - 38.7 mg/L of GLGPG for 24 h and proteins were quantified by multiparametric analysis (see Materials and Methods). Only the effects of 250 mg/L RHP and 9.7 mg/L GLGPG are shown.

^a ^protein secreted by stimulated/protein secreted by unstimulated cells

^b ^*p *value = significance value between (LPS/IFN-γ)- and (LPS/IFN-γ + substance) -treatment.

**Table 4:** Effect of RHP and GLGPG on gene expression in human PBL

Gene	C_T_^a^	LPS/IFN-γ	LPS/IFN-γ + RHP (250 mg/L)		LPS/IFN-γ + GLGPG (9.7 mg/L)	
		fold change	fold change	*p *value^b^	fold change	*p *value^b^
COX-2	22.8	**8.6**	**8.6**	0.880	**6.8**	0.16
TNF-α	24.9	**16.1**	**8.9**	0.004	**12.4**	0.04
IL-1α	27.2	**17.9**	**11.3**	0.002	**16.1**	0.37
IL-6	29.8	**54.8**	**43.6**	0.254	**55.6**	0.94
						
CCL5/RANTES	25.8	**1.2**	**1.2**	0.908	**1.3**	0.45
CXCL10/IP-10	35.5	**1444**	**190**	< 0.001	**2340**	0.04
MIP-2	28.6	**16.8**	**18.7**	0.577	**15.6**	0.66
MIP-3α	28.7	**37.6**	**32.3**	0.159	**23.1**	0.01
						
5-LOX	25.5	**0.8**	**0.6**	0.018	**0.8**	0.96
MMP-9	27.3	**0.8**	**0.9**	0.830	**0.8**	0.81

LPS/IFN-γ -stimulated cells were cultured with 31.3 - 1000 mg/L RHP or 1.2 - 38.7 mg/L of GLGPG for 2 h and gene expression was quantified by RT-PCR. Fold changes were calculated as indicated in Materials and Methods. Only the effects of 250 mg/L RHP and 9.7 mg/L GLGPG are shown.

^a^C_T _= cycle threshold (C_T _< 25 = high basal gene expression; 25 < C_T _< 30 = intermediate basal gene expression; C_T _> 30 = low basal gene expression).

^b^*p *value = significance value between (LPS/IFN-γ)- and (LPS/IFN-γ + substance) -treatment

### Rose hip and GLGPG modulate catabolic gene expression in chondrosarcoma SW1353 cells

The chondrosarcoma SW1353 cell line was used as a surrogate for primary chondrocytes. In response to exogenous IL-1β, these cells altered the expression of a similar set of genes as primary chondrocytes [28] (Tables 5 and 6). IL-1β activated SW1353 cells displayed increases in gene expression levels of MMP-1, -3, -13 and ADAMTS-4. In contrast, MMP-2 and ADAMTS-5 were barely affected. Treatment of IL-1β-activated SW1353 cells with RHP or GLGPG led to a significant inhibition of gene expression of MMP-1, -3, -13 but not of ADAMTS-4 or MMP-2 (Table 5). IL-1β treatment only slightly influenced expression levels of anabolic genes (*i.e*. aggrecan, collagen), whereas other mediators such as COX-2, TNF-α, iNOS, IL-6 and leukemia inhibitory factor (LIF) [14,29] were markedly altered by IL-1β stimulation (see fold changes in Table 5). Basal chemokine gene expression was low in SW1353 cells; IL-1β treatment induced dramatic increases of chemokine mRNA levels, with CCL5/RANTES and MIP-3α being the most responsive. RHP significantly decreased the expression levels of five chemokine genes (MIP-2, MIP-3α, IL-8, CCL5/RANTES and CXCL10/IP-10) (Table 5) and cytokine genes including IL-1α, IL-1β and IL-6. Whereas GLGPG significantly reduced CXCL10/IP-10 expression, it augmented that of MIP-2 or IL-8. This feature was also observed with regard to anabolic genes, where GLGPG, in contrast to RHP, increased aggrecan and collagen expression. It should be stressed that the chosen *in vitro *conditions preferably trigger catabolic activity in chondrocytes, while anabolic events would have been favored at different culture conditions.

**Table 5:** Effect of RHP and GLGPG on gene expression in human chondrosarcoma SW1353 cells

Gene	C_T_^a^	IL-1β	IL-1β + RHP (250 mg/L)		IL-1β + GLGPG (9.7 mg/L)	
		fold change	fold change	*p *value^b^	fold change	*p *value^b^
MMP-1	27.2	6.3	3.6	0.002	4.5	0.019
MMP-2	22.3	0.8	0.6	0.006	1.0	0.142
MMP-3	32.7	182.7	80.6	< 0.001	137.0	0.003
MMP-9	31.8	6.3	7.1	0.285	10.7	0.001
MMP-13	28.4	17.7	8.9	< 0.001	12.7	0.005
ADAMTS-4	35.3	10.7	9.8	0.136	9.6	0.026
ADAMTS-5	31.1	0.8	0.7	0.476	1.4	0.006
TIMP-1	21.2	1.4	1.2	0.195	1.9	0.007
						
Aggrecan	32.0	1.8	1.3	0.017	3.7	< 0.001
Collagen I	19.2	1.1	0.8	0.028	1.8	0.001
COL2A1	31.9	0.8	0.6	0.001	0.9	0.526
						
MIP-2	40.2	1169	474	< 0.001	1626	0.006
MIP-3α	38.5	19642	12001	< 0.001	20822	0.437
CCL5/RANTES	42.9	35920	27081	0.048	41476	0.353
IL-8	34.5	12022	5599	< 0.001	16308	0.001
CXCL10/IP-10	39.7	3274	836	0.001	1436	0.001
						
IL-1α	37.7	42.3	29.9	0.036	32.0	0.065
IL-1β	31.9	135.3	118.1	0.195	100.8	0.012
IL-6	38.4	10846	5969	0.001	8876	0.122
IL-1Ra	31.9	1.1	1.4	0.381	1.7	0.111
IL-1RI	27.1	1.7	0.9	0.001	1.7	0.885
						
TNF-α	34.5	319.2	705.1	0.001	567.8	0.003
iNOS	35.4	17.6	22.4	0.139	21.2	0.236
COX-2	34.4	23.4	19.0	0.080	35.3	0.005
LIF	30.1	64.8	53.9	0.118	42.1	0.005

IL-1β -stimulated cells were cultured with 31.3 - 1000 mg/L RHP or 1.2 - 38.7 mg/L of GLGPG for 4 h and gene expression was quantified by RT-PCR. Fold changes were calculated as indicated in Materials and Methods. Only the effects of 250 mg/L RHP and 9.7 mg/L GLGPG are shown.

^a^C_T _= cycle threshold (C_T _< 22 = high basal gene expression; 23 < C_T _< 30 = intermediate basal gene expression; C_T _> 30 = low basal gene expression).

^b^*p *value = significance value between (IL-1β)- and (IL-1β + substance) -treatment.

**Table 6:** Effect of RHP and GLGPG on gene expression in NHAC-kn cells

Gene	C_T_^a^	IL-1β	IL-1β + RHP (250 mg/L)		IL-1β + GLGPG (9.7 mg/L)	
		fold change	fold change	*p *value^b^	fold change	*p *value^b^
MMP-1	29.5	22.8	10.4	0.008	20.0	0.418
MMP-2	23.4	0.8	0.6	0.249	0.8	0.332
MMP-3	28.1	167	89	0.048	128	0.135
MMP-9	29.6	7.2	6.0	0.025	5.0	0.203
MMP-13	28.1	2.2	1.3	0.048	2.0	0.135
ADAMTS-4	29.5	20.0	21.7	0.460	14.6	0.103
ADAMTS-5	21.7	1.3	0.9	0.011	1.7	0.148
TIMP-1	17.2	1.2	0.9	0.052	1.1	0.215
Aggrecan	28.8	1.0	0.8	0.196	1.1	0.340
Collagen 1	18.0	0.7	0.5	0.05	0.8	0.30
COL2A1	22.2	1.0	0.5	0.137	1.2	0.325
MIP-2	29.9	441	110	0.082	626	0.147
MIP-3α	30.0	481	271	0.007	436	0.126
CCL5/RANTES	26.7	87	42.0	0.009	54	0.046
IL-8	26.3	799	168	0.002	760	0.356
CXCL10/IP-10	nd^c^					
IL-1β	31.3	1078	530	0.002	505	0.085
IL-6	29.8	2011	1048	0.069	1927	0.226
IL-1Ra	28.4	1.2	0.6	0.107	1.2	0.272
IL-1RI	22.6	0.9	0.5	< 0.001	0.8	0.301
TNF-α	nd^c^					
iNOS	nd^c^					
COX-2	26.58	133	48.3	0.11	186	0.03
LIF	23.8	35.2	19.2	0.003	28.9	0.239

IL-1β -stimulated cells were cultured with 31.3 - 1000 mg/L RHP or 1.2 - 38.7 mg/L of GLGPG for 4 h and gene expression was quantified by RT-PCR. Fold changes were calculated as indicated in Materials and Methods. Only the effects of 250 mg/L RHP and 9.7 mg/L GLGPG are shown.

^a^C_T _= cycle threshold (C_T _< 22 = high basal gene expression; 23 < C_T _< 30 = intermediate basal gene expression; C_T _> 30 = low basal gene expression).

^b^*p *value = significance value between (IL-1β)- and (IL-1β + substance) -treatment

^c^nd = not done

### Modulation of catabolic gene expression in primary human articular chondrocytes

Normal human articular chondrocytes from knee (NHAC-kn) were activated by IL-1β in the presence of increasing concentrations of RHP or GLGPG. Basal expression levels of the monitored genes were comparable in SW1353 and NHAC-kn except for ADAMTS-5, collagen 2A1, four chemokine genes and IL-6, which were more expressed in NHAC-kn (Tables 5 and 6). The IL-1β induced changes in NHAC-kn gene expression levels were as marked as those observed in SW1353 cells. IL-1β significantly up-regulated catabolic genes (ADAMTS-4, MMP-1 and MMP-3), interleukins (IL-1α, IL-1β, IL-6), chemokines (IL-8, CCL5/RANTES, MIP-2 and MIP-3α) and COX-2 (Table 6). LIF experienced strong up-regulation by IL-1β, a feature that was not shared by the receptor for IL-1 (IL-1RI) and IL-1 receptor antagonist (IL-1Ra). The patho-physiological stimulus *(i.e*. IL-1β) weakly affected anabolic genes like collagen 1, collagen 2 and aggrecan (Table 6).

**Figure 3:**
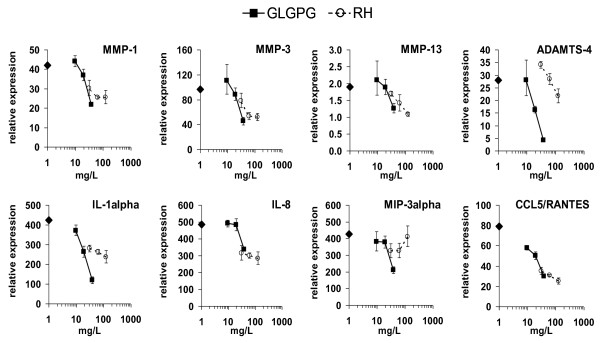
**Effect of RHP and GLGPG on gene expression in chondrocytes**. IL-1β-stimulated NHAC-kn were cultured for 4 h with 62.5 - 250 mg/L RHP or 9.7 - 38.7 mg/L of GLGPG and expression of the indicated genes was quantified by RT-PCR. Symbols on the y axis indicate the gene expression in IL-1β stimulated NHAC-kn (relative to unstimulated NHAC-kn).

RHP significantly diminished the expression level of MMP-1, MMP-3, MMP-9 and MMP-13, but had only moderate effects on ADAMTS-4, TIMP-1 or ADAMTS-5 (Table 6). Similarly, MIP-2, MIP-3α, CCL5/RANTES and IL-8 were down-regulated by up to 75%. The effects on IL-1β, IL-1RI, and IL-6 were also statistically significant. In contrast, GLGPG weakly influenced mRNA levels of most of the tested genes (*p *value ~0.1). The effects of the substances were concentration-dependent (Figure 3). It should be noted that RHP had more potent effects on gene expression in NHAC-kn than in PBL (Figure 2). Anabolic genes were barely altered at 4 h of IL-1β stimulation.

### Distinct reactivity of different cell types to RHP and GLGPG

**Figure 4:**
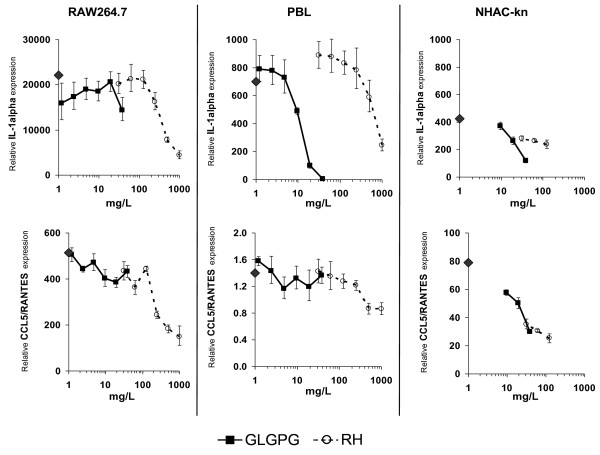
**Comparison of the effects of RHP and GLGPG on murine macrophages, human PBL and chondrocytes**. The symbols on the y-axis indicate gene expression levels in stimulated cells in the absence of substances. Cells were cultured for 4 h (RAW264.7; NHAC-kn) or 2 h (PBL).

Murine macrophages, human PBLs and chondrocytes revealed idiosyncratic patterns of reactivity to RHP and GLGPG as exemplified for IL-1α and CCL5/RANTES (Figure 4). Treatment of cells with LPS and IL-1β, respectively, elicited tissue-specific responses; IL-1α was induced in all three cell populations. Concomitantly, the effect of test compounds was robust with RHP being more potent than GLGPG. Conversely, CCL5/RANTES induction was strong in RAW264.7 cells and NHAC-kn but virtually absent in PBL. In general, RHP and GLGPG had effects on genes that were strongly activated by IL-1β or LPS; conversely, non-induced genes were virtually unaffected.

## Discussion

In this study, a panel of biological properties of rose hip powder and its constituent galactolipids, GLGPG, has been described for the first time and provides evidence that cellular parameters related to cartilage destruction and inflammatory responses were modulated by these natural compounds. This feature has been established using two approaches: (1) effects on inflammatory processes including cytokines and chemokines were monitored in macrophages and peripheral blood leukocytes, (2) modulation of catabolic activity and the production of chemokines and cytokines were determined in SW1353 cells and NHAC-kn *in vitro*.

An adequate homeostasis between anabolic and catabolic events ensures tissue rebuilding and renewal in intact cartilage [1]. Growth factors including insulin-growth factors, connective-tissue growth factors, TGF-β or BMP favor proliferation and differentiation of chondrocytes and the synthesis of collagen and aggrecan. Conversely, inflammatory factors (*e.g*. pro-inflammatory interleukins, eicosanoids and nitric oxide) and proteinases (*e.g*. MMPs and ADAMTS) induce tissue erosion and degrade ECM components, respectively. MMP-1 and -13 preferably cleave type II collagen [4]. MMP-3 has broader substrate specificity; ADAMTS-4 and -5 cleave proteoglycans [30]. IL-1β is the most important inducer of catabolic processes in OA. TNF-α, IL-6 and LIF also contribute to tissue erosion in advanced stages of OA, although their implication in rheumatoid arthritis (RA) prevails [31]. IL-1β activated primary chondrocytes express catabolic factors that degrade the ECM. OA chondrocytes were rather refractory to the patho-physiological trigger [7,32]. Here, we show that RHP and GLGPG impaired IL-1 expression in macrophages, leukocytes and chondrocytes. LIF, which is also involved in OA [14,28,29], was reduced by RHP and GLGPG. The data suggest that the substances exert their effects at an early phase of OA development and target different cell populations. Admittedly, this hypothesis needs to be tested in appropriate preclinical models.

While IL-6 is of key importance in RA, it is also involved in OA [33-36]. RHP inhibited IL-6 gene expression in chondrocytes, whereas its impact on cytokine production in macrophages or leukocytes was marginal. Thus, the effect of compounds was confined to cells, where excessive IL-6 production was deleterious; conversely, IL-6 was not modulated in peripheral blood leukocytes where it is required for an efficient humoral immune response. Similarly, TNF-α has multiple actions in the pathogenesis of RA [31] and may be a contributing factor to OA [1]. The tested natural substances reduced its expression in macrophages and leukocytes, while in chondrosarcoma cells the opposite effect on gene expression was observed. The meaning of this dichotomy is unclear and requires further investigation. Collectively, changes in IL-6 and TNF-α expression by RHP and GLGPG could influence the etiology of OA and RA. Indeed, in a recent clinical study, dietary supplementation of RA patients with RHP alleviated RA symptoms [37]. Obvious limitations in the interpretation of the current *in vitro *results should be noted: (1) the absence of data on bioavailability and pharmacokinetics of the substances makes it difficult to correlate the described *in vitro *effects with *in vivo *efficacy, (2) additional clinical trial to assess efficacy (anti-inflammatory *versus *pain-relief) of the test substances are warranted, (3) a possible effect of RHP and GLGPG on joint space widening should be investigated.

The involvement of NO and PGE_2 _in OA has been described in numerous studies [11,38-43]. The enhanced production of NO in OA joints contributed to a slowly progressing inflammation [9]. IL-1β treatment of chondrocytes induced iNOS and concomitant expression of cartilage-degrading enzymes [41,42,44-46]. Conversely, the progression of murine OA was slowed down in iNOS knock-out mice [47]. NO also activated MMPs [40] and PGE_2 _production [48] with concomitant inhibition of proteoglycan and collagen synthesis [43,49]. Chondrocyte apoptosis was promoted by NO and PGE_2 _[8,50-52]. In view of the observation that RHP and GLGPG diminished NO and PGE_2 _production, they might have anti-apoptotic effects in chondrocytes. The consequence of this inhibition is pivotal, since it affects (1) survival of chondrocytes, (2) production of pro-inflammatory cytokines, and (3) activation of ECM-degrading enzymes. At the molecular level, the expression of prostaglandin E_2 _synthase (*i.e*. mPGES) [53-57] rather than COX-2 was altered by RHP (Table 2) and therefore only weakened the production of pro-inflammatory prostaglandins.

MMP-1, MMP-3, MMP-13, ADAMTS-4 and ADAMTS-5 are key catabolic enzymes that degrade collagen and proteoglycan; their sequential expression might occur at, and herald different phases in the progression of OA [28,32,58]. Increased expression of tissue inhibitor of matrix metalloproteinase (TIMP) is associated with remodelling of articular tissues [59]. IL-1β induced a high MMP-1 expression in primary chondrocytes, which reflects events related to early OA; SW1353 displayed a pattern of MMP expression that relates to intermediate stages of OA. RHP and GLGPG exerted effects only on a sub-set of these enzymes: MMP-1, -3 and -13 and ADAMTS-4. Other members of the MMP family that have a role in tissue remodeling (*e.g*. MMP-9) [60] were not influenced by these treatments.

The impact of chemokines in OA has been substantiated previously [61]: CCL5/RANTES and CXCL8/IL-8 were identified in activated chondrocytes or OA tissue [14-16,62]. Expression levels of chemokines and their receptors dramatically change in IL-1β activated chondrocytes [7]. This emphasizes the putative role of chemokines in early and intermediate phases of progressing OA. Given the described effects of RHP and GLGPG, it is tempting to hypothesize that RHP components act as biological modifiers on chemotaxis in OA chondrocytes. In accordance with the *in vitro *study, clinical trials have provided evidence that chemotaxis of leukocytes is reduced after dietary supplementation with RHP [17].

Biological modulators such as IL-1β or NO eventually activate MAPK that, in turn, leads to the translocation of NF-κB to the nucleus and NF-κB dependent gene activation. The kinetics and extent of RelA and NF-κB1 expression follow similar kinetics and amplitude in IL-1β stimulated SW1353, primary chondrocytes [28] and RAW264.7 cells (this study). *In vitro *studies have further demonstrated that this pathway was modulated by various substances contained in the food chain [63-68]. Regulatory motifs identified in chemokine genes include NF-κB [69]. Direct evidence that RHP and GLGPG may act along this pathway is provided by the observed down-regulation of NF-κB1, NF-κB49, NF-κBp65 and - as a consequence of decreased re-synthesis - I-κBα in activated macrophages (Table 2). The analysis of modification of these transcription factors like phosphorylation is required to substantiate this hypothesis. As previously shown [7,52,70,71], binding elements for other transcription factors (MEF-3, AP-1 and CEBPβ) have been mapped to the regulatory region of IL-1β- or LPS-responsive genes [7] and might also interact with RHP and GLGPG.

The *in vitro *effects described in this study were elicited at high concentrations of RHP and GLGPG. To date, no bioavailability studies have been reported for RHP; but it is unlikely that IC_50 _values for RHP and its constituent bioactive components are achieved in the body fluids or tissues as a consequence of dietary uptake. It is possible that after dietary intake RHP constituents accumulate in peripheral blood leukocytes and thus locally achieve threshold concentrations required for biological effects. Assuming that GLGPG is the only bioactive component in RHP powder, it can be deduced from the IC_50 _values given in Table 1 that RHP needs to contain ~3% of GLGPG. Yet, since the GLGPG contents of the studied RHP preparation does not exceed 0.1% of the dry plant mass (our unpublished results), we hypothesize that other substances contribute to the biological activity of RHP. The observation that substances contained in the food chain alter features of chondrocyte biology has been documented previously: polyphenols, including resveratrol and catechins (epigallocatechin-3-gallate, EGCG) reduced the expression of MMP-1, -3 or -13 and modulated levels of iNOS and COX-2 [63,65-68,72-74].

## Conclusions

Both the onset and development of OA is expected to be modulated by the effects of RHP and GLGPG: (1) the observed diminished NO and IL-1β production is likely to delay or prevent initial steps of the disease, (2) the homeostasis of anti- and pro-inflammatory cytokines is also modulated and thus provides a means to attenuate inflammatory processes in OA, and (3) chemokines that predominantly direct the migration of neutrophils were less abundantly produced (Table 7). RHP and its constituents thus modulate cellular and molecular processes that may explain the positive effect of RHP observed in clinical trials. The effects on interleukin and chemokine production as well as MMP expression indicate that RHP and its constituents down-regulate catabolic processes and reduce chemotaxis related to OA or RA. Collectively, the data provide a molecular and biochemical basis for the cartilage protection by RHP.

**Table 7:** Synopsis of effects of RHP and GLGPG on cellular processes

Type of mediators	Effect of RHP and GLGPG			
	
	*In murine macrophages*	*In human PBL*	*In chondrocytes*	*Possible consequences of reduced expression or production*
**Chemokines** (*chemokine family*)				
MIP-1α (*CC*)	Not significant	Reduced by GLGPG	-^a^	Recruitment of monocytes, T cells, B cells and eosinophils reduced by GLGPG
MIP-1β (*CC*)	-^a^	Reduced by GLGPG	-^a^	Recruitment of monocytes and neutrophils reduced by GLGPG
MIP-3α (*CC*)	-^a^	Reduced	Reduced	Recruitment of T lymphocytes reduced
CCL5/RANTES (*CC*)	Not significant	Reduced	Reduced	Recruitment of leukocytes diminished
				
MIP-2 (*CXC*)	-^a^	Not significant	Reduced by RHP	Recruitment of neutrophils diminished by RHP
IL-8 *(CXC*)	-^a^	Reduced	Reduced by RHP	Mainly reduced recruitment of neutrophils
CXCL10/IP-10 (*CXC*)	Reduced	Reduced	-^a^	Recruitment of activated T cells diminished
				
**Interleukins/cytokines**				
IL-10	Increased	Increased	-^a^	Enhancement of anti-inflammatory processes
IL-1α	Reduced	Reduced	Reduced	Attenuation of inflammatory processes
IL-1β	Reduced	Not significant	Reduced	Attenuation of inflammatory processes
IL-6	Unchanged	Unchanged	Reduced by RHP	Modulation of inflammatory processes in OA and RA
TNF-α	Reduced	Reduced	-^a^	Effects on initiation and progression of RA
				
**Growth & differentiation factors**				
G-CSF	Unchanged	Increased by RHP	Not significant	Modulation of immune response
GM-CSF	Unchanged	Increased by RHP	Not significant	Modulation of immune response
VEGF	-^a^	Increased by RHP	Not significant	Promotes angiogenesis
IFN-γ	Unchanged	Reduced	Not significant	Altered immune response

^a^- = not measured

## List of abbreviations

ADAMTS: a disintegrin and metalloproteinase with thrombospondin type I motif; GLGPG: galactolipid (2*S*)-1: 2-di-*O*-[(9*Z*: 12*Z*: 15*Z*)-octadeca-9: 12: 15-trienoyl]-3-*O*-β-D-galactopyranosyl glycerol; IL: interleukin; LPS: lipopolysaccharide; NO: nitric oxide; MMP: matrix metalloproteinase; OA: osteoarthritis; PBL: peripheral blood leukocytes; PGE_2_: prostaglandin E_2_; RA: rheumatoid arthritis; RHP: rose hip powder.

## Competing interests

This research was funded by DSM Nutritional Products, where all authors are currently employed.

## Authors' contributions

JS and NR conceived the experiments, NR performed the experiments, JS and NR analysed the experimental data, UH provided analytical data, JS and NR have written the paper, SW participated in drafting the study and revising the data. All authors have read and approved the final manuscript.

## Pre-publication history

The pre-publication history for this paper can be accessed here:

http://www.biomedcentral.com/1472-6882/11/105/prepub

## Supplementary Material

Additional file 1 **Effects of RHP and GLGPG on gene expression in murine macrophage cell line RAW264.7**. RAW264.7 cells were stimulated with LPS and cultured with 250 mg/L RHP or 9.7 mg/L of GLGPG for 4 h and gene expression was quantified by RT-PCR. Fold changes were calculated as specified in Materials and Methods. Click here for file

Additional file 2 **Effects of RHP and GLGPG on cytokine/chemokine production in human peripheral blood leukocytes**. LPS/IFN-γ -stimulated peripheral blood leukocytes were cultured with 250 mg/L RHP or 9.7 mg/L of GLGPG for 24 h and proteins were quantified by multi-parametric analysis as described in Materials and Methods. Click here for file

Additional file 3 **Effects of RHP and GLGPG on gene expression in human peripheral blood leukocytes**. LPS/IFN-γ -stimulated cells were cultured with 250 mg/L RHP or 9.7 mg/L of GLGPG for 2 h and gene expression was quantified by RT-PCR (for details see: Materials and Methods). Click here for file
