# Supplementary material for: Rose hip and its constituent galactolipids confer cartilage protection by modulating cytokine, and chemokine expression
Source: BMC Complement Altern Med. 2011 Nov 3;11:105. doi: 10.1186/1472-6882-11-105 (PMC3231956; doi:10.1186/1472-6882-11-105)

### Additional Material file

### Schwager et al. ‘Rose hip and calactolipids confer cartilage protection by modulation of interleukin, cytokine and chemokine Expression’

**Additional file 1:**

**Effects of RHP and GLGPG on gene expression in murine macrophage cell line RAW264.7**

RAW264.7 cells were stimulated with LPS and cultured with 250 mg/L RHP or 9.7 mg/L of GLGPG for 4 h and gene expression was quantified by RT-PCR. Fold changes were calculated as indicated in Materials and Methods.

###
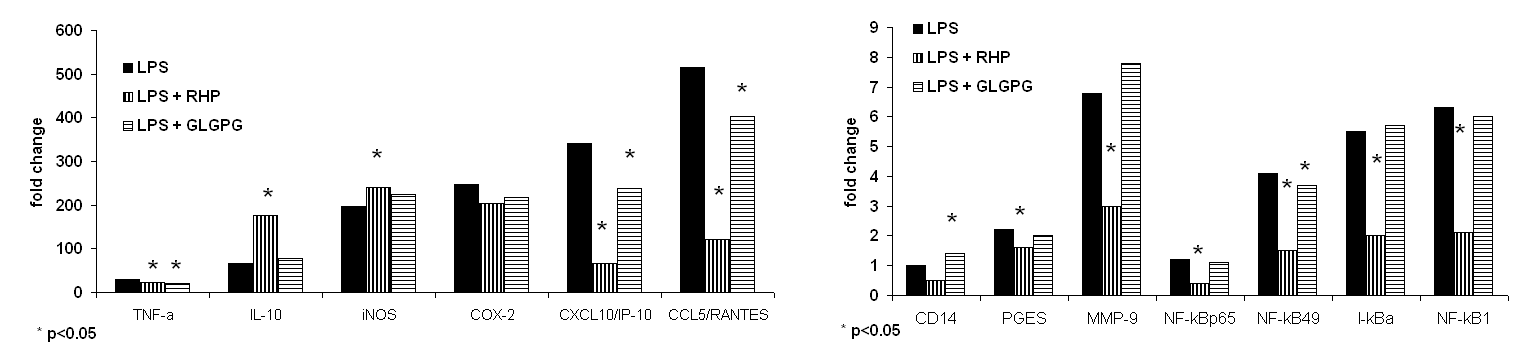

Supplement: Additional file 1 — Effects of RHP and GLGPG on gene expression in murine macrophage cell line RAW264.7. RAW264.7 cells were stimulated with LPS and cultured with 250 mg/L RHP or 9.7 mg/L of GLGPG for 4 h and gene expression was quantified by RT-PCR. Fold changes were calculated as specified in Materials and Methods. [file 1472-6882-11-105-S1.DOC]
