# Supplementary material for: Rose hip and its constituent galactolipids confer cartilage protection by modulating cytokine, and chemokine expression
Source: BMC Complement Altern Med. 2011 Nov 3;11:105. doi: 10.1186/1472-6882-11-105 (PMC3231956; doi:10.1186/1472-6882-11-105)

### Additional Material file

### Schwager et al. ‘Rose hip and calactolipids confer cartilage protection by modulation of interleukin, cytokine and chemokine Expression’

**Additional file 2:**

**Effects of RHP and GLGPG on cytokine/chemokine production in human peripheral blood leukocytes**

LPS/IFN-γ -stimulated peripheral blood leukocytes were cultured with 250 mg/L RHP or 9.7 mg/L of GLGPG for 24 h and proteins were quantified by multi-parametric analysis (see Materials and Methods).


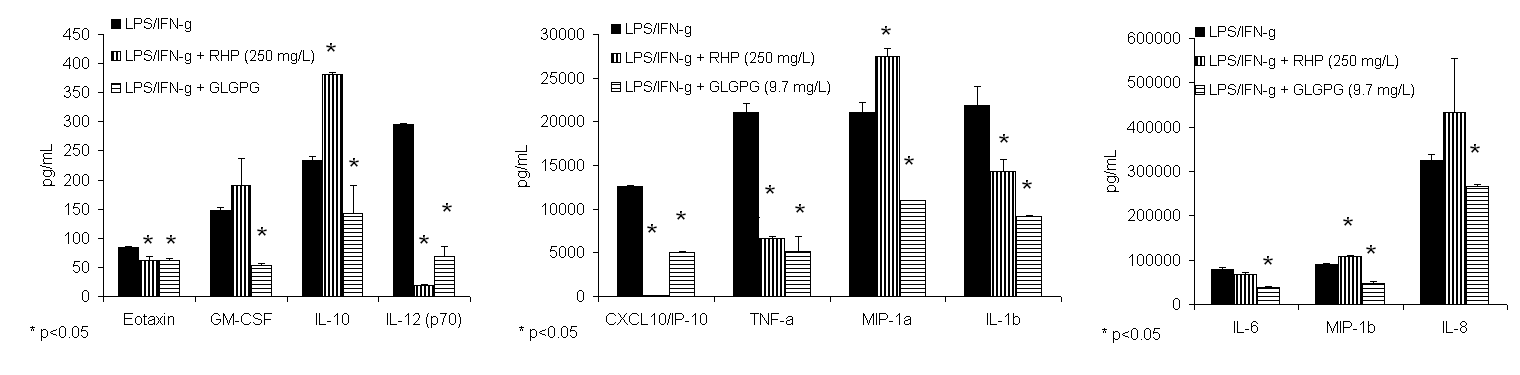

Supplement: Additional file 2 — Effects of RHP and GLGPG on cytokine/chemokine production in human peripheral blood leukocytes. LPS/IFN-γ -stimulated peripheral blood leukocytes were cultured with 250 mg/L RHP or 9.7 mg/L of GLGPG for 24 h and proteins were quantified by multi-parametric analysis as described in Materials and Methods. [file 1472-6882-11-105-S2.DOC]
