# Supplementary material for: Rose hip and its constituent galactolipids confer cartilage protection by modulating cytokine, and chemokine expression
Source: BMC Complement Altern Med. 2011 Nov 3;11:105. doi: 10.1186/1472-6882-11-105 (PMC3231956; doi:10.1186/1472-6882-11-105)
Supplement: Additional file 3 — Effects of RHP and GLGPG on gene expression in human peripheral blood leukocytes. LPS/IFN-γ -stimulated cells were cultured with 250 mg/L RHP or 9.7 mg/L of GLGPG for 2 h and gene expression was quantified by RT-PCR (for details see: Materials and Methods). [file 1472-6882-11-105-S3.DOC]

### Additional Material file

### Schwager et al. ‘Rose hip and calactolipids confer cartilage protection by modulation of interleukin, cytokine and chemokine Expression’

**Additional file 3:**

**Effects of RHP and GLGPG on gene expression in human peripheral blood leukocytes**

LPS/IFN-γ -stimulated cells were cultured with 250 mg/L RHP or 9.7 mg/L of GLGPG for 2 h and gene expression was quantified by RT-PCR (see Materials and Methods).

**
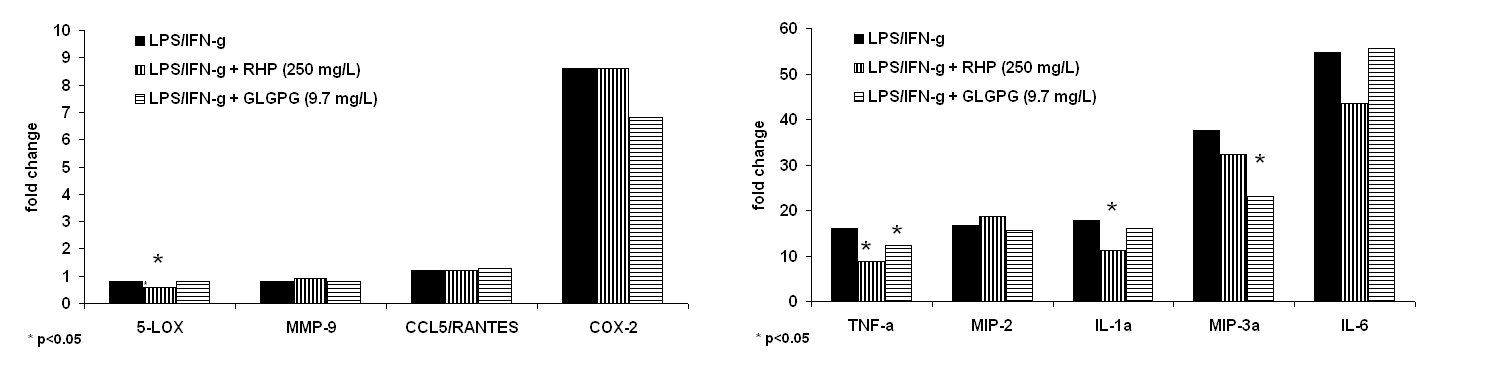
**
